# Response of Tonsil Follicular Dendritic Cell Sarcoma to Multimodal Treatment Including Pembrolizumab: A Case Report and Literature Review

**DOI:** 10.3389/fonc.2022.816903

**Published:** 2022-03-01

**Authors:** Nanxiang Chen, Wei Ba, Dawei Zhao, Lei Sheng, Xinxin Zhang

**Affiliations:** ^1^ College of Otolaryngology Head and Neck Surgery, Chinese PLA General Hospital, Chinese PLA Medical School, National Clinical Research Center for Otolaryngologic Diseases, State Key Lab of Hearing Science, Ministry of Education, Beijing Key Lab of Hearing Impairment Prevention and Treatment, Beijing, China; ^2^ Department of Pathology, Chinese PLA General Hospital, Chinese PLA Medical School, Beijing, China; ^3^ Department of Radiation Oncology, Chinese PLA General Hospital, Chinese PLA Medical School, Beijing, China; ^4^ Department of Anesthesiology, Zhejiang Provincial People’s Hospital, Affiliated People’s Hospital, Hangzhou Medical College, Hangzhou, China

**Keywords:** follicular dendritic cell sarcoma, programmed cell death 1 ligand (PD-L1), pembrolizumab, immune check inhibitor (ICI), multimodal treatment

## Abstract

Follicular dendritic cell sarcoma (FDCS) is a rare malignant neoplasm that was classified by the World Health Organization (WHO) under histiocytic and dendritic cell neoplasms in the 2016 revision. Considering the rarity of this tumor, there is no standardized treatment. It is usually treated by complete surgical resection. Adjuvant chemotherapy and radiotherapy are alternative methods. Immune checkpoint inhibitors (ICIs) represented by the programmed death receptor 1/programmed death ligand 1 (PD-1/PD-L1) antibody have achieved significant clinical benefits in a variety of solid tumors. However, reports on the treatment of FDCS with ICIs are rare. FDCS often expresses high levels of PD-L1, which provides a rationale to use immunotherapy in cases of FDCS. Here, we present a 51-year-old Filipino-Chinese man with FDCS who was treated with multimodal treatment, including the PD-1 inhibitor pembrolizumab and achieved a relatively long disease-free survival of 24 months. This case emphasizes that the application of ICIs under the guidance of NGS technology seems to be a meaningful treatment option for patients with FDCS.

## Introduction

Follicular dendritic cell sarcoma (FDCS) is an extremely rare stromal tumor. The European Network for Rare Cancers reported that the crude incidence of FDCS was 0.05 per 100000 per year (1). It mainly occurs in extranodal sites (79.4%). Lymph nodes are involved in only approximately 15% of cases (2). Those with an extranodal origin in the head and neck region are extremely rare. The tonsil is the most common site of FDCS in the head and neck. The first case of tonsillar FDC tumor was reported in 1986 (3). Other affected sites include the spleen, liver, gastrointestinal tract, skin, lungs, mediastinum, and soft tissues (3–6).

Considering the rarity of this tumor, there is no defined standard treatment for cases of inoperable and metastatic DFCS. For locally advanced and inoperable FDCS, comprehensive treatment, including radiotherapy and chemotherapy, can be selected. However, these treatments are palliative, and the treatment response is not good. Immune checkpoint inhibitors (ICIs), especially programmed death-1 (PD-1)/programmed death factor ligand-1 (PD-L1), have made breakthrough progress in various cancers, bringing survival benefits to patients (7–9). However, there are currently no case reports describing patients with FDCS who progressed after the CHOP regimen and achieved a longer disease-free survival after receiving ICIs alone.

Here, we present a case of tonsillar FDCS with neck lymph node metastasis receiving an ICI (pembrolizumab) as part of therapy for metastatic disease, achieving a PFS as second-line treatment of 24 months and demonstrating that such therapy is a potential treatment option for patients with FDCS. This report also provides a brief review of the previous literature on the treatment of FDCS.

## Case Presentation

**Figure 1:**
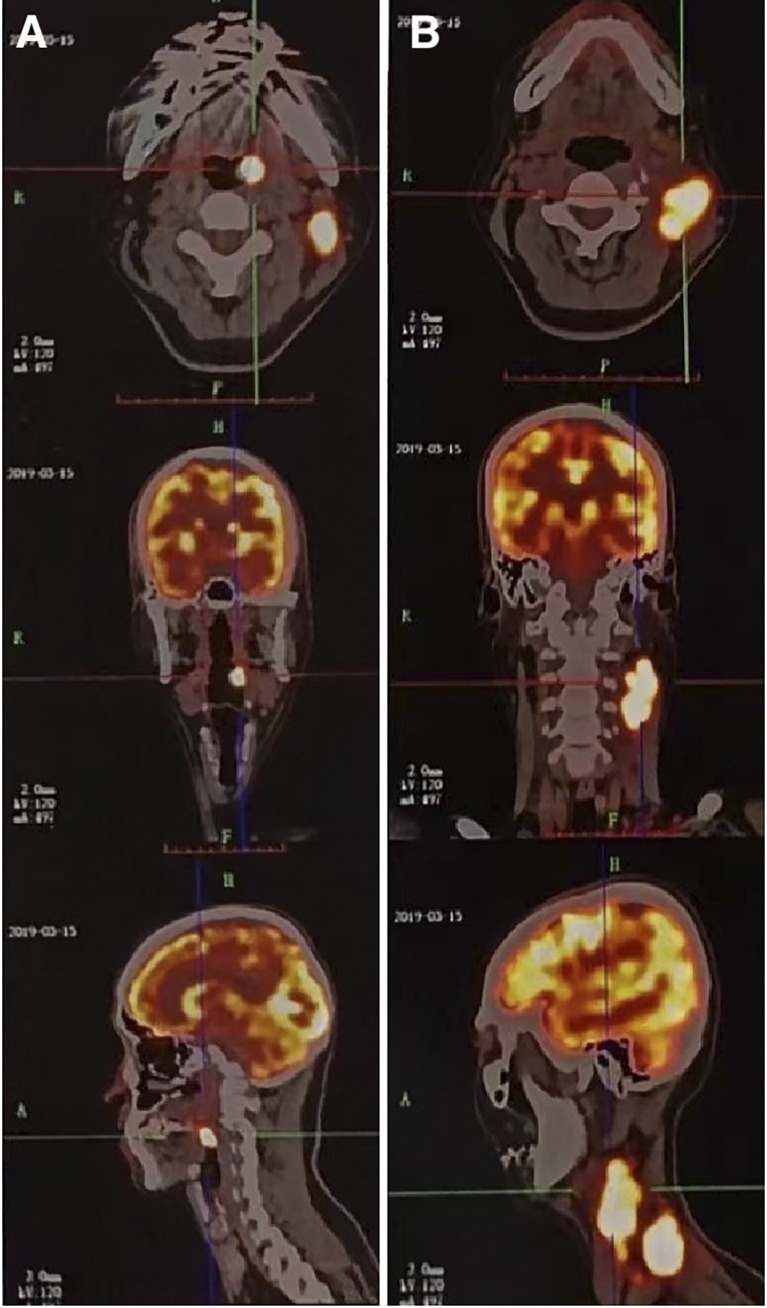
A 51-year-old male patient with tonsillar follicular dendritic cell sarcoma (FDCS). **(A)** PET/CT scan shows extensive hypermetabolic activity on the left side of the oropharynx (SUVmax 17.7). **(B)** PET/CT scan shows extensive hypermetabolism in the left neck II, III, IV, and Vb lymph nodes (SUVmax 14.1). CT, computed tomography; PET, positron emission tomography; SUVmax, standardized uptake value maximum.

**Figure 2:**
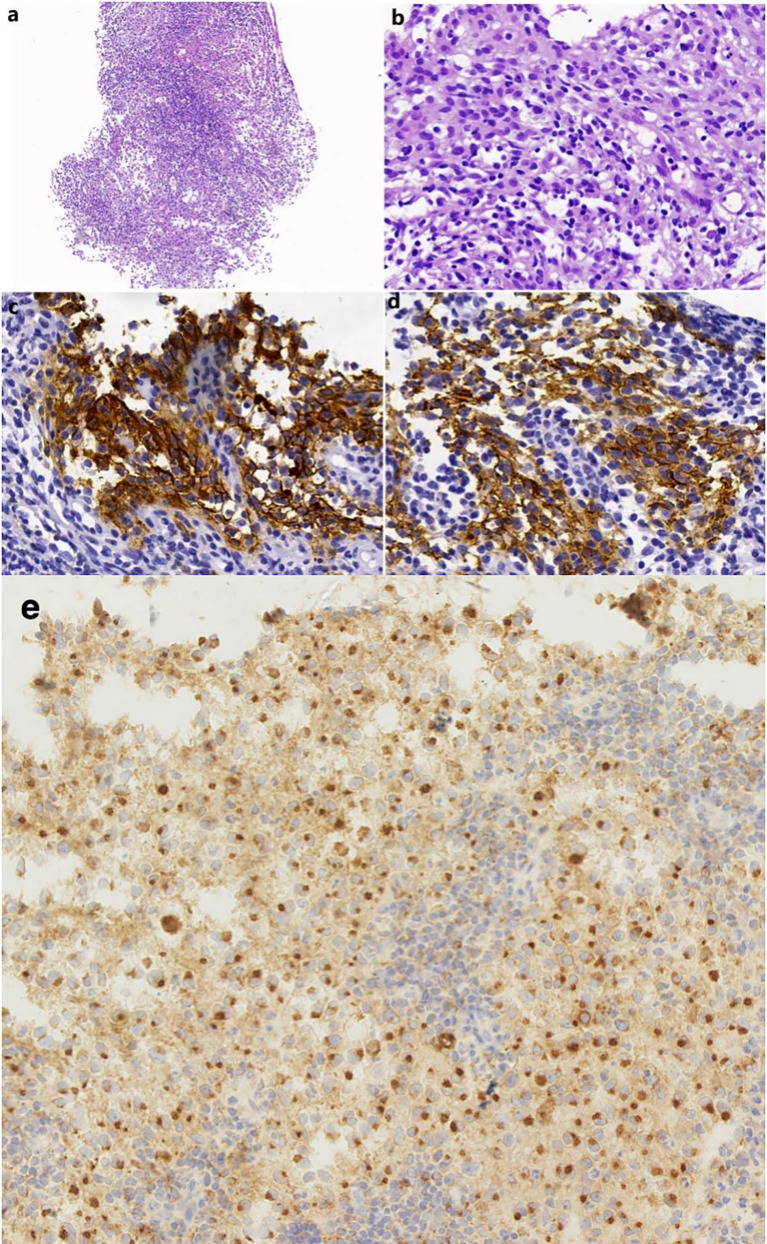
**(A)** A needle biopsy from the left neck mass shows nodular or focal infiltrate of tumor cells with mixed lymphocytes and neutrophils (HE, original magnification 100×). **(B)** The tumor cells contain rich and lightly stained cytoplasm and vesicular nuclei (HE, 400×). **(C, D)** The tumor cells were positive for CD21 (400×) and CD35 (400×). **(E)** The tumor cells were positive for CXCL13 (400×).

**Figure 3:**
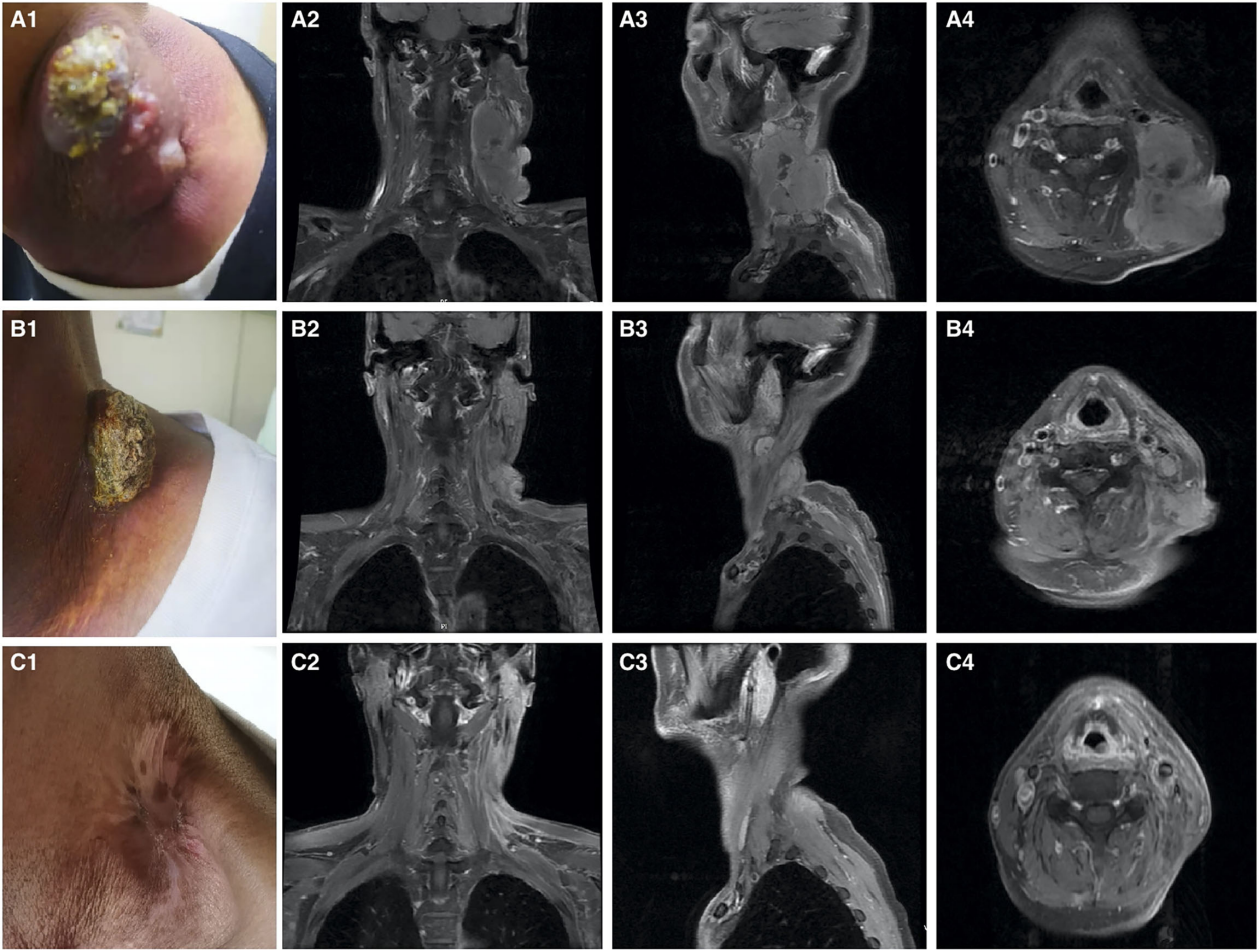
Imaging of the patient on treatment. **(A1–A4)** Pretreatment. **(B1–B4)** After chemoradiotherapy. **(C1–C4)** After 3 cycles of immunotherapy. **(A1)** Solid mass in the left neck. **(A2–A4)** MRI showing a large mass lesion in the left neck with liquefactive necrosis. **(B1)** Tumor is significantly reduced. **(B2–B4)** MRI evaluation. The left neck tumor is significantly reduced, but residual shadows are still visible. **(C1)** The neck mass disappears. **(C2–C4)** MRI evaluation. The left neck tumor and the lymph nodes under the armpit are not palpable.

A 51-year-old man presented with a solid mass approximately 10 cm x 7 cm x 5 cm in size in the left neck, which was accompanied by afternoon and night fevers, night sweats, and a maximum body temperature of 39.5 °C. The solid mass had unclear borders and cauliflower-like surfaces with purulent secretions attached (**Figure 3A1**). He went to the hospital on March 15, 2019. A PET/CT scan showed extensive hypermetabolic activity in the left side of the oropharynx, the lower edge of the left tonsil (SUVmax 17.7, **Figure 1A**) and the left neck II, III, IV, and Vb lymph nodes (SUVmax 14.1, **Figure 1B**). The tumor was positive for CD21, CD35 and CXCL13 and negative for cytokeratin and CD23. The Ki-67 proliferation index was 20% (**Figure 2**). MRI revealed a large uneven mass on the left side of the neck, which was accompanied by liquefaction and necrosis and was squeezing and surrounding the internal jugular arteries and veins (**Figures 3A–C**). According to the results of pathology and imaging, specialists from three hospitals rendered a consensus diagnosis of tonsillar FDCS with neck lymph node metastasis.

After the consultation by head and neck surgeons, it was determined that the tumor could not be completely removed by surgery, so a combination treatment of radiotherapy and chemotherapy was given to the patient. After one cycle of cyclophosphamide, doxorubicin, vincristine, and prednisone (CHOP), partial remission was observed. However, the patient experienced grade III liver toxicity and grade IV hematological toxicity that required administration of recombinant human granulocyte stimulating factor. When the patient’s hematological toxicity and liver function returned to normal, another cycle of induction chemotherapy (modified CHOP: vindesine, 3 mg/m^2^/day 1; liposomal doxorubicin, 20-25 mg/m^2^/days 1~2; ifosfamide, 2.5 g/m^2^/days 1~3 and methylprednisolone, 80 mg/days 1~5) and concurrent chemoradiotherapy were given to the patient. Radiotherapy treatment consisted of irradiation of the tumor volume at 2.09 Gy/fraction in 33 fractions (total RT dose 69 Gy) with concomitant liposomal doxorubicin (20 mg/m^2^ on day 1 and day 8, 21 days/cycle) After concurrent chemoradiotherapy, the tonsil mass disappeared, and the tumor in the left neck was significantly reduced (**Figures 3B1–B4**). However, a new 2.7 cm x 2.1 cm lymph node was palpable under the left armpit, and ultrasound indicated that it was a “metastatic lymph node.”

**Figure 4:**
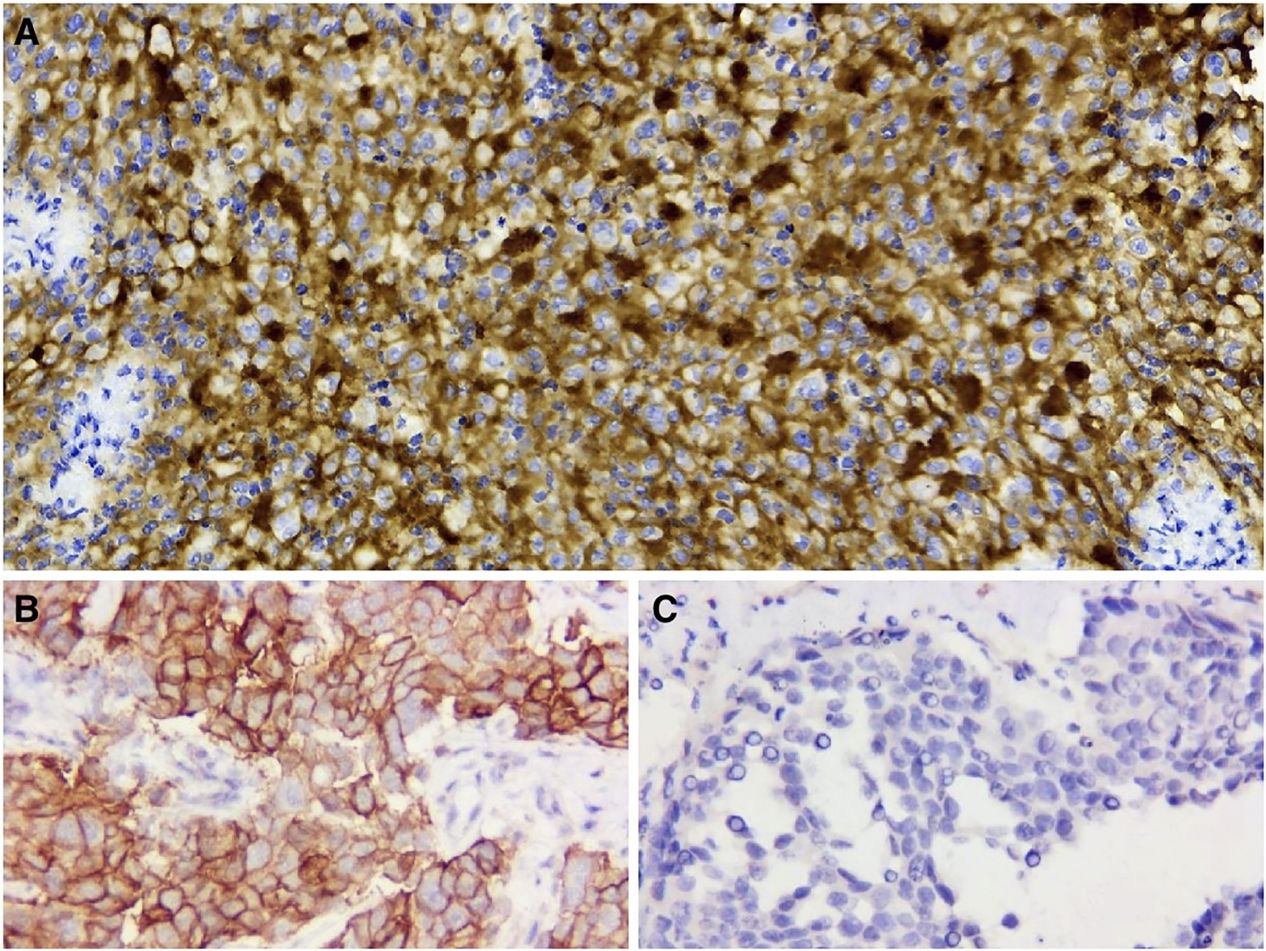
PD-L1 immunohistochemical staining (400×). **(A)** PD-L1 staining is positive (TPS 95% and CPS 95). **(B)** Positive control. **(C)** Negative control.

To assist in further treatment decisions, commercial next-generation sequencing (NGS) and PD-L1 immunohistochemical tests were performed. NGS helps us identify tumor-related somatic mutations, which can provide a reference for tumor-targeted therapy. At the same time, through the assessment of tumor mutation burden (TMB), PD-L1 expression, and tumor microsatellite instability (MSI), we can obtain more comprehensive reference information related to tumor immunotherapy. Exons of 457 cancer-related genes were detected (**Supplemental Table 1**), while no actionable mutations were found (**Supplemental Table 2**). PD-L1 staining was positive (**Figure 4**, details of antibodies shown in **Supplemental Table 3**), the tumor proportion score (TPS) was 95%, the combined positive score (CPS) was 95, and the tumor mutational burden (TMB) was 6.13/Mb. According to the high expression of PD-L1, the patient began to take pembrolizumab, a PD-1 inhibitor. He received pembrolizumab 200 mg every 3 weeks (Q3 W) in the first 3 cycles. After 3 cycles of immunotherapy, complete remission was observed in the patient’s neck mass (**Figures 3C1–C4**), the lymph nodes under his arm could not be palpated, and his body temperature returned to normal. Because oropharyngeal mucositis caused by radiotherapy affected the patient’s oral feeding, the patient’s body weight continued to decrease in the late stage of radiotherapy and 1 month after the end of radiotherapy. When he returned to the Philippines, his weight dropped to 53 kg. In the treatment of melanoma, pembrolizumab is effective at a dose of 2 mg/Kg. Therefore, considering that the patient has achieved CR after three cycles of immunotherapy, and the body weight has decreased significantly, the dose of pembrolizumab in the next six cycles was adjusted to 100 mg.

A follow-up PET/CT scan (November 20, 2019) showed mildly increased FDG uptake in the left cervical lymph node (SUV 2.3) and a 3 × 2 cm high metabolic nodule in the left upper lobe of the lung near the pleura (SUV 6.8). Considering that pulmonary nodules may be inflammatory nodules, an anti-inflammatory drug was used, and the nodules successfully regressed during the 4-month follow-up period. The patient received a total of 9 cycles of immunotherapy. PET/CT showed no obvious abnormalities, and the disease had not yet progressed.

## Discussion

FDCS originates from follicular dendritic cells located in the germinal center of lymph nodes, where they present antigens to B cells (10, 11). About 7%-10% of cases are associated with hyaline vessel Castleman disease (2). Because FDCS can occur in many different primary sites and its heterogeneity is obvious, FDCS can often be confused with tumors such as large cell lymphomas (12), melanoma, gastrointestinal stromal tumor, and leiomyoma. Morphologically, FDCS often appear as spindle-shaped or spindle-shaped cells with indistinct cell borders. Pathological diagnosis often requires immunohistochemical staining for multiple FDC markers. In addition, histiocytic and dendritic cell neoplasms are sometimes misdiagnosed as sarcomas because of their mesenchymal origin and complex karyotypes that behave like low-grade soft tissue sarcomas (13). Histologically, FDCS often presents as spindle to ovoid cells characteristically arranged in storiform arrays or whorled patterns. Usually, they are dual-core or occasional multicore forms (10). Follicular dendritic cell sarcoma (FDCS) is positive for one or more FDC markers (e.g., CD21, CD35 and CD23). CXCL13 and podoplanin (D2~40) are usually positive but not completely specific. It is negative for CD1a, CD34, lysozyme, and MPO. The Ki-67 proliferation index is 1-25% (mean: 13%) (10).

The molecular landscape of FDCS is not well described. Recurrent loss-of-function alterations involved in the negative regulation of NF-κB activation (*NFKBIA, CYLD*) and cell cycle progression (*CDKN2A, RB1*) were observed (14). The *BRAF V600E* mutation has been reported in some cases of FDCS (15), and BRAF inhibitors may have a beneficial effect on the treatment of these tumors. For this patient, detection of immune-related genes was included in NGS (shown in **Supplemental Table 4**). No negative regulators of immunotherapy, such as STK11, JAK1/2, PTEN, or genes related to hyperprogressive disease (HPD), such as MDM2/4, were found, which meant that he had no absolute contraindication to immunotherapy.

Considering the rarity of FDCS, there is no standardized treatment. It is usually treated by complete surgical resection. The local recurrence rate and distant metastasis rate are 28% and 27%, respectively (16). Chemotherapy and radiotherapy are alternative treatments, but both are controversial. CHOP (cyclophosphamide, doxorubicin, vincristine, and prednisone), which is frequently used for lymphoma treatment, has been used to treat FDCS. A study reported that patients with liver FDCS have been successfully treated with surgery and CHOP. However, the size of the tumor did not significantly reduce, and it showed decreased FDG avidity and increased necrosis in the tumor (17). Jain et al. reported that gemcitabine with a taxane (commonly adopted in some soft tissue sarcoma subtypes) yielded an overall response rate of 80% in patients with FDCS (18). In a report of two patients with FDCS metastatic to the liver, gemcitabine and docetaxel achieved a durable partial objective response (19). Radiotherapy also plays a key role in the treatment of FDCS. Adjuvant radiotherapy can improve local control of the disease (18, 20). Eun et al. reported a case of FDCS benefitting from adjuvant radiotherapy and achieved a 2-year disease-free period (21).

In recent years, immune checkpoints have become a hot spot in the field of cancer treatment research. Immune checkpoint inhibitors (ICIs) represented by the programmed death receptor 1/programmed death ligand 1 (PD-1/PD-L1) antibody have achieved significant clinical benefits in a variety of solid tumors. Among lymphomas, classic Hodgkin’s lymphoma (cHL) and primary mediastinal large B-cell lymphoma have been approved for indications. According to the reports, PD-L1 staining was positive in 50-80% of FDCS cases, which provided a rationale to use immunotherapy in patients with FDCS (13, 22, 23). Copy-number gain on chromosome 9p24 containing CD274 (PD-L1) and PDCD1LG2 (PD-L2) has been observed in FDCS (14). Agaimy et al. reported that 54% of 13 assessable cases showed moderate to strong membranous staining for PD-L1 (13). Gatalica et al. observed overexpression of PD-L1 in all three patients with FDCS that were examined (24). Xu et al. reported that 50% (10 of 20) of FDCS cases were positive for PD-L1, and 55% (11 of 20) of cases were also positive for PD-L2 (25). A study on the histogenesis and immunological microenvironment based on FDCS showed conspicuous reactivity for PD-L1 in more than 60% of cases, whereas in other mesenchymal tumors, only 4% of cases displayed detectable PD-L1 (26). All these studies indicate that ICIs may be an option for the treatment of FDCS. However, there are few reports about patients with FDCS receiving treatment with ICIs (**Supplemental Table 5**). Cingam et al. reported a case of a patient with FDCS who had liver metastases and received nivolumab treatment, which was not successful (27). In 2020, two more patients (retroperitoneal FDCS and pelvic FDCS) were reported. They received nivolumab and ipilimumab and had evidence of tumor responses (23). Lei et al. described a patient with primary small intestine FDCS who received sintilimab plus lenvatinib as third-line treatment and achieved a PFS of 7 months (25).

Here, we report one patient with different clinical presentations. A 51-year-old man diagnosed with FDCS of the tonsil with neck lymph node metastasis who received induction chemotherapy followed by concurrent chemoradiotherapy. After concurrent chemoradiotherapy, the tonsil mass disappeared, and the tumor was significantly reduced. However, MRI showed a new lymph node under the left armpit. After confirming by NGS that the patient had no immune-related negative genes, had high PD-L1 expression, and had high CPS and TPS, a PD-1 inhibitor, pembrolizumab (Keytruda), was then given, and after 6 cycles of immunotherapy, The patient’s neck mass is not palpable, the lymph nodes under his arm could not be palpated, and his body temperature returned to normal. The patient no longer continues to use immunotherapy and other anti-tumor treatments, and has achieved a high quality of life. The disease has not progressed since follow-up. Although TMB was low in this case, immunotherapy could still show efficacy in the presence of high TPS and CPS.

## Conclusion

To our knowledge, this is the first reported case of tonsillar FDCS treated with induction chemotherapy, concurrent chemoradiotherapy and ICIs. Compared with other case reports, this patient achieved a relatively long disease-free survival period. This case suggests that patients who have progressed after the CHOP regimen may be able to obtain a good treatment response by using ICIs alone. This emphasizes that the application of ICIs under the guidance of NGS technology seems to be a meaningful treatment option for patients with FDCS. Of course, the therapeutic effect of ICI monotherapy for such patients with FDCS is still unknown. A large-scale prospective clinical trial is needed to gain more experience with these drugs in these diagnostic families.

## Data Availability Statement

The original contributions presented in the study are included in the article/**Supplementary Material**. Further inquiries can be directed to the corresponding author.

## Ethics Statement

The studies involving human participants were reviewed and approved by Ethics Committee of Chinese PLA General Hospital. The patients/participants provided their written informed consent to participate in this study.

## Author Contributions

NC, DZ, and XZ treated the patient. DZ performed the radiotherapy in this case. WB analyzed the pathological information. NC and WB drafted the case report. NC and LS performed the literature review. All authors contributed to the article and approved the submitted version.

## Conflict of Interest

The authors declare that the research was conducted in the absence of any commercial or financial relationships that could be construed as a potential conflict of interest.

## Publisher’s Note

All claims expressed in this article are solely those of the authors and do not necessarily represent those of their affiliated organizations, or those of the publisher, the editors and the reviewers. Any product that may be evaluated in this article, or claim that may be made by its manufacturer, is not guaranteed or endorsed by the publisher.

## Supplementary Material

Supplementary Table 1 457 cancer-related genes.

Supplementary Table 2 SNVs and InDels.

Supplementary Table 3 Details of antibodies.

Supplementary Table 4 Immune-related genes included in NGS.

Supplementary Table 5 Previous reports of FDCS treated with ICI.
