# Supplementary material for: Response of Tonsil Follicular Dendritic Cell Sarcoma to Multimodal Treatment Including Pembrolizumab: A Case Report and Literature Review
Source: Front Oncol. 2022 Mar 1;12:816903. doi: 10.3389/fonc.2022.816903 (PMC8922364; doi:10.3389/fonc.2022.816903)
Supplement: Supplementary Table 5 — Previous reports of FDCS treated with ICI. [file Table_5.docx]

| study | No. of cases | Prior systemic therapy | ICI Treatment | outcome |
| --- | --- | --- | --- | --- |
| Cingam SR, 2017 | 1 case | CHOP every 21 days | nivolumab (3 mg/kg every 21 days) | CHOP: local control in the mediastinum, but rapidly progress in the liver  ICI: failure |
| Lee MY, 2020 | 2 cases | Case 1 VAdrC  Case 2 None | ipilimumab/nivolumab induction therapy and continued on Nivo monotherapy. | stable disease after 8–12 weeks of starting treatment |
| Lei Y, 2021 | 1 case | 8 cycles of CHOP  6 cycles of ABVD | sintilimab and lenvatinib | 7 months PFS |
| Our case | 1 case | CHOP+Radiotherapy | Pembrolizumab | 24 months PFS |

Supplemental Table 5. Previous reports of FDCS treated with ICI
